# Operationalizing Agile Methods: Examining Coherence in Large-Scale Agile Transformations

**DOI:** 10.1007/978-3-030-58858-8_8

**Published:** 2020-08-18

**Authors:** Noel Carroll, Finn Olav Bjørnson, Torgeir Dingsøyr, Knut-Helge Rolland, Kieran Conboy

**Affiliations:** 6 IT University of Copenhagen, Copenhagen, Denmark; 7 University of British Columbia, Vancouver, BC Canada; 8 Lero, National University of Ireland Galway, Galway, Ireland; 9 Norwegian University of Science and Technology, Trondheim, Norway; 10 SINTEF Digital, Trondheim, Norway; 11 University of Oslo, Oslo, Norway

**Keywords:** Large-scale agile transformation, Normalization Process Theory, Coherence, Case study, Organizational change, Autonomous teams

## Abstract

Following the highly pervasive and effective use of agile methods for software development, attention has now turned to the much more difficult challenge of applying these methods in large scale, organization-wide development. However, identifying to what extent certain factors influence success and failure of sustaining large-scale agile transformations remains unclear and there is a lack of theoretical frameworks to guide such investigations. By adopting Normalization Process Theory and specifically ‘coherence’, we compare two large-scale agile transformation case studies and the different perspectives individuals and teams had when faced with the problem of operationalizing the agile method as part of their large-scale agile transformation. The key contributions of this work are: (i) this is a first attempt to present the results of a comparison between a successful and failed large-scale agile transformations; and (ii) we describe the challenges in understanding the rationale, differences, value, and roles associated with the methods to support the large-scale agile transformation. We also present future research for practitioners and academics on large-scale agile transformation.

## Large-Scale Agile Transformation

Agile methods have been well received by practitioners and academics over the past two decades. Given the success of agile approaches at the team level, many large software organizations have begun to scale these methods to a large-scale and often enterprise-wide context [1]. We adopt the description of “transformation” from [2] which explains how the concept of transformation and “scaling up” are very closely related to describing how development organizations with small agile practices (e.g. a single agile team in a large setting) scale their agile practices to at least 50 people or 6 teams (i.e. large-scale agile practices). However, such large-scale adoption has proven challenging [3, 4], with very few successful cases reported across literature which hampers the research community in learning about specific factors of large-scale agile transformation processes. The literature identifies particular challenges such as the complexity and uncertainty introduced when a method tries to enable radical and continuous change across a fragmented set of teams and projects across an organization [2], the confusion caused by numerous variants and misinterpretations of that method [5], as well as the limitations of both top-down (management-driven) or bottom-up (team-driven) agile method transformations [2, 5]. The objective of this study is to explore coherence as one key part of normalization and in operationalizing large-scale agile transformations. Coherence is the process of sensemaking that individuals and organizations undergo in order to promote or inhibit the routine embedding of a practice (i.e. determining specifically “what is the work?”). We achieve this by (i) comparing coherence across two separate case studies (a successful and failed large-scale agile transformation); (ii) reporting on the key lessons learned around the need to consider coherence of a large-scale agile transformation; and (iii) presenting a summary of recommendations for organizations on scaling agile methods as a continuum rather than a change in state which the notion of transformations can imply.

## Normalization Process Theory

Normalization Process Theory (NPT) is a derivative sociological theory on the implementation, embedding and integration of new technologies and organizational innovations [6] which allows us to challenge assumptions around embedding change during transformations [7]. NPT provides a rich theoretical lens to explain a transformation process since it allows us to uncover whether practices become routinely embedded in their social contexts as the result of people working, individually and collectively, to enact them. There are four main NPT constructs which explain normalization:

1. **Coherence:** the meaningful qualities of a specific practice
2. **Cognitive participation:** enrolment and engagement of individuals and groups
3. **Collective action:** interaction with already existing practices
4. **Reflexive monitoring:** how a new practice is understood and assessed by implicated actors

Each NPT construct comprises of four theoretical components, i.e. 16 components in total [7]. Within each of the core theoretical constructs, we can examine the normalization of large-scale agile transformations and shed new insights on organizing structures, social norms, group processes and conventions, i.e. work relating to assessing patterns of work and outcomes. NPT provides practical insights on specific phenomena both qualitatively and quantitatively such as examining the sustainability of large-scale agile methods [7]. NPT allows us to unpack the dynamic nature of large-scale agile transformations by focusing on the social organization of the work (implementation) of making practices routine elements of everyday life (embedding), and of sustaining embedded practices in their social context (integration). For the large-scale agile transformation case study comparison in this study, we focus on the coherence. We focus on coherence because it uncovers four key components of initiating and operationalizing new practices:

1. **Differentiation:** Comparing differences in an old and new set of practices
2. **Communal specification:** Building a shared understanding of the vision, aims, objectives, and expected benefits of a set of practices.
3. **Individual specification:** Assessing individual perceptions on their specific tasks and responsibilities around a new set of practices.
4. **Internalization:** Evaluating team members perception on the value, benefits, and importance of a new set of practices.

Specifically, coherence is a critical stage of large-scale agile transformations as it enables us to focus on sensemaking carried out individually and collectively when faced with the problem of operationalizing a set of practices, i.e. in this research context, operationalizing a large-scale agile transformation method. This also allows us to examine the rationale and drivers to transform an organization’s practices and compare how people make sense of (re)defining and (re)organizing practices.

## Research Method

Comparative case studies involve the analysis and synthesis of the key similarities, differences and emerging patterns across two or more cases [8]. This method is suitable to explore new topic areas which focus on ‘how’ or ‘why’ questions around a contemporary set of events.

This study compares two large-scale agile transformation projects using the FinanceCo [7] and PublicOrg [9] case studies. In the context of this research, both cases share a common research objective in understanding an organization’s experience in undertaking a large-scale agile transformation (Table 1). Both cases were selected based on a specific criteria [2] in that the organizations with small agile practices scales their agile practices to at least 50 people or 6 teams (i.e. large-scale agile practices). In the context of the case studies, both qualitative and quantitative methods were adopted to better understand the context which influences the success and failure of a transformation process.

**Table 1.** Comparative case study summary

Description	FinanceCo	PublicOrg
Sector	Financial Services	Public Services
Employees	50,000	20,000
Locations	USA, Ireland, India, China	Norway
Agile method (before transformation)	Customized	Customized (based on [10])
Large-scale agile method	Spotify	Multidisciplinary semi-autonomous teams
Result	Method abandoned after two years in favor of SAFe	Method in use with good results
Study timeframe of transformation	2017–2018	2016–2020
No. of interview participants	8 development teams (50 participants)	10 development teams (39 participants)
Data collection method(s)	Semi-structured interviews; observations; access to systems, documents, reports, meetings	Semi-structured interviews; observations; access to systems, documents, reports, meetings

## Findings

This section presents a summary of the key findings on the four components of coherence to explain efforts on the normalization of the two large-scale agile transformations. We compare how each of these components were operationalized and contributed to the success and failure of normalizing a large-scale agile transformation.

### Differentiation

By examining *differentiation*, we can compare how FinanceCo and PublicOrg managed the initial stages of the large-scale transformation. For FinanceCo, there was no clear evidence that their teams attempted to differentiate the new practices associated with the large-scale agile transformation strategy. Management considered high-level differences and used sweeping claims to promote the potential of the Spotify model in terms of outcomes. Management presented ideas around how the Spotify model would address some ongoing business challenges, for example through a new software development culture, fluid team structures, and continuous software development flow. However, Squads were tasked with operationalizing the Spotify model with little guidance or expectation on how to differentiate the new practice to old ways of working.

In contrast, PublicOrg demonstrated evidence of a planned induction period to inform all stakeholders on the implications and expectations from the large-scale agile transformation. This included both consultants from two different suppliers and the developers from PublicOrg. More concretely a work group consisting of representatives working on business needs, software architecture, and development recommended to change deployment model from a bimodal model to a model of multidisciplinary semi-autonomous teams. The group delivered a 24-slide presentation to the project manager with a joint proposal, which sought to align development practice in the project with a future way of working in PublicOrg. The group considered the deployment model after identifying dependencies, suitability for continuous deployment, cross-functional teams, time criticality and user value.

### Communal Specification

By focusing on *communal specification*, we compared how teams built a shared understanding of the vision, aims, objectives, and expected benefits of the large-scale agile transformation. Within FinanceCo, Squads perceived that the Spotify model had imposed changes to divisional structures and created a separation of powers. However, management had reported that team restructuring was imposed in an effort to build and sustain relational work through self-organized teams and autonomy to drive change. For FinanceCo, the overall objective to transform was to improve software team productivity and performance (guided by software flow metrics). However, a Squad Lead viewed the Spotify model as a way to remove predictability of team performance and control across relational work: *“It’s difficult to make sense of the Spotify model. You want certainty, predictivity, and control on the management side. Yet, you adopt the Spotify model because you have admitted that you don’t want predictability or control for the transformation process.”*

PublicOrg, however, launched a four-month subproject prior to introduction of the new deployment model in order to “*build competence on agile methods*” in the project. The main vision described by PublicOrg was to transition from a delivery model based on phases and handovers to a flow-based model where the division between customer and supplier is invisible. The change involved end-to-end automatic testing, toggling of features, one shared stream of code, and an improved deployment pipeline. The change project aimed to minimize “*work in progress*” and to establish a code base per product to minimize complexity of development. The expected benefits described in the report included “higher quality and user value”, “earlier realizations of business value”, “more time to develop solution, less time on reporting and documentation”, “a more motivating workday for employees”. PublicOrg also hired two agile coaches to assist in the transition process. One of the Technical Leads described how: *“The agile coach we had* – *without him the whole process would have been a lot more painful!”.*

### Individual Specification

By focusing on *individual specification*, we compare how individuals across both case studies perceived their specific tasks and responsibilities imposed by the large-scale agile method. From a managerial perspective at FinanceCo, the Spotify model provided a roadmap for the roles and responsibilities required to improve organizational-wide agility and software team performance and productivity. However, there was a lack of clarity in terms of how it would be operationalized and it was not always well received by software developers. As one software developer within the Platform Chapter of a Squad at FinanceCo explained: *“not only are we forced to change roles but now we are held to account to reach new performance targets in these roles…”.*

At PublicOrg, a work group was established and recommended: “*The degree of autonomy must be adapted to each domain, based on need of collaboration, dependencies, and the connection to the central administrative system team, and this might change over time*.” Preparation for a new model started early, a product owner stated, “*I was aware that this change was coming since I was assigned to the project in 2017. So, the first thing I did was to attend one of those two*-*days agile workshops. That was two months before I started this project*.” A functional advisor experienced a varying clarity of roles: “*The thing is, my role is very vaguely defined. The product owner, that role is very specified, you attribute a lot of responsibility to that role, really. Too much if you ask me, and then me and another is in the role of functional advisor which is very vaguely defined, just supporting the product owner, really*.

### Internalization

For *internalization*, we explored team members perception on the value, benefits, and importance of the new agile method. Within a FinanceCo transformation context we probed whether value, benefits, and importance related to topics such as financial, business, cultural, or personal. However, the concepts of ‘value’, ‘benefits’, and ‘importance’ were considered to be a vague or elusive from both a management and team perspectives and efforts were placed on developing metrics to represent how work should be prioritized. A Senior Business Intelligence Developer in the Business Intelligence Chapter stated: *“Our progress or lack of progress is probably best reflected in the amount of unplanned work we are faced with which makes it difficult to understand the value of using the Spotify model.”*

PublicOrg, on the other hand, present evidence of more optimism around the transformation process and their transition using a large-scale agile method. For example, a Technical Lead (previous Scrum Master) explains: *“It’s not really much of a difference. I see it as a good idea, and it’s good to have shorter decision paths”* In addition, a Tester at PublicOrg described their experience by stating: *“Summing up the transition, I’m happy that we transitioned to such an agile way of working. It makes my workday easier and more fun, if I’m allowed to say that. Less stressful and I feel more ownership and responsibility for the functionality we deliver as a team.”*

## Discussion

This research focuses on coherence as a critical stage of large-scale agile transformations and compares how two organizations faced the problem of operationalizing a new set of practices. Table 2 presents a summary of our comparative findings. We summarize how FinanceCo had a relatively weak foundation and attempted to adopt a “*scale and learn approach*”. This approach was largely based on many weak assumptions around operationalizing the Spotify model which eventually led to growing tensions across the organization and failure in their transformation efforts. In contrast, we learned how PublicOrg had a strong foundation and implemented an agile culture and adopted a “*learn and scale approach*” which proved to be very successful in operationalizing the reorganization into autonomous teams. The key contribution of examining coherence is that we identify tensions between management expectations and teams operationalizing new practices.

**Table 2.** Summary of comparative findings on operationalizing large-scale agile methods

Coherence	FinanceCo	PublicOrg
Differentiation	Weak foundation in terms of competencies in place and poor communication to differentiate the transformation method and goals	Strong foundation in terms of competencies in place and excellent communication to differentiate the transformation method and goals
	Lack of agile training prior to transformation to instill an agile culture and mindset	Planned induction of agile training provided prior to transformation and implementation of an agile culture
Communal specification	Sense of imposed changes to divisional structures and created a separation of powers	Introduced a project to build competence on agile methods
	Improvements on software team productivity and performance were guided by software flow metrics	Recruitment of two agile coaches to assist in the transition process
Individual specification	Method provided a roadmap for the roles and responsibilities required to improve organizational-wide agility and software team performance and productivity	Emphasis on need of collaboration, dependencies, and the connection to the central administrative system team
	Lack clarity across teams on how the new method would be operationalized in practice	Awareness of change brought about by the methods a number of years prior to the transformation
Internalization	Concepts of ‘value’, ‘benefits’, and ‘importance’ were considered to be a vague or elusive from both the management and team perspectives	Evidence of value in large-scale transformation, e.g. shorter decision paths
	Efforts placed on developing metrics to represent how work should be prioritized	Improved sense of satisfaction in an agile way of working and more ownership and responsibility as a team

We identify how complexity and uncertainty emerge when organizations try to instigate change across an organization [2] due to the lack of clarity on a large-scale transformation process and weak assumptions on how to manage the process. We uncover some of the key tensions which go unreported throughout literature regarding the top-down (management-driven) or bottom-up (team-driven) agile method transformations [2, 5]. Our findings indicate the need for organizations to become more proactive by introducing a large-scale agile transformation induction period to embed an agile culture and mindset across the organization before undergoing any transformation process. While communication is often documented as a generic yet key factor for large-scale agile transformation throughout literature [10], we identify the need to specifically focus on coherence in order to compare differences in an old and new agile method and to have a shared understanding of the vision, aims, objectives, and expected benefits of a large-scale agile transformation.

By focusing on coherence, we also identify the importance of providing dedicated resources to support the transformation, for example, agile coaches being available at team level to understand how to operationalize a scaling agile method. There is little comparative research on how organizations assess individual perceptions of specific tasks and responsibilities around a large-scale agile transformation. This research demonstrates how critical it can be to evaluate team members perception of a large-scale agile transformation at the very early stages [5].

## Conclusion

We present two clear contributions from this research: (i) present the results of a comparison between a successful and failed large-scale agile transformations; (ii) we describe the challenges in understanding the rationale, differences, value, and roles associated with the methods to support and sustain a successful large-scale agile transformation and factors which contribute to failed transformations. While we adopt NPT to focus on one of the theoretical constructs of coherence, we present future research for practitioners and academics on large-scale agile transformation on the NPT framework which explains how practices become implemented, embedded, integrated, and evaluated. As part of our future research, we will focus on comparing additional cases on assumptions associated with large-scale agile transformations. NPT could set new directions for research on large-scale agile development [3], agile transformation [4], and extending into other research developments on IT-enabled and digital transformations [11].
